# Developmental and reproductive toxic effects of exposure to microplastics: A review of associated signaling pathways

**DOI:** 10.3389/ftox.2022.901798

**Published:** 2022-08-31

**Authors:** Itishree Dubey, Sabbir Khan, Sapana Kushwaha

**Affiliations:** ^1^ Department of Pharmacology and Toxicology National Institute of Pharmaceutical Education and Research, Raebareli (NIPER-R), Transit Campus, Raebareli, India; ^2^ Department of Neuro-Oncology The University of Texas, MD Anderson Cancer Center, Houston, TX, United States

**Keywords:** BTB-blood–testis barrier, MPs-microplastics, developmental toxicity, signaling, reproductive toxicity

## Abstract

Microplastics (MPs), small pieces of plastic (∼5 mm), are released into the environment not only as a result of the decomposition of large-sized plastics but also from day-to-day use of plastic products. Chronic exposure to MPs has been attributed to harmful effects on aquatic organisms and rodents. Effects include gastrointestinal toxicity, hepatotoxicity, neurotoxicity, and reproductive and developmental toxicities. Exposure to MPs may also potentially affect human health. Herein, we reviewed the impact of MPs on male and female reproductive systems and the associated mechanisms involved in the reproductive and developmental toxicities of MPs. We performed a literature search in Google Scholar and PubMed using the following keywords: MPs and reproductive toxicity; MPs and developmental studies; MPs and infertility; MPs and aquatics; and MPs and rodents. Evidence of MPs accumulation has been reported in many organs of humans and experimental models. The harmful effects of MPs have been manifested in male and female reproductive systems of mammalian and aquatic animals, including developmental effects on gametes, embryos, and their offspring. This review describes various signaling pathways involved in MPs-associated male and female reproductive and developmental toxicities.

## Introduction

Plastics are frequently used in day-to-day life due to their low cost, ease of handling, transportation, production process, and widespread applications. The production of plastics has been increasing continuously for the last 60 years. Many of these are broken down into small plastics called microplastics (MPs) (Avio et al., 2017). Mechanical stress, sunlight, and an oxidizing atmosphere decompose large plastics into MPs, typically 5 mm in diameter. Numerous samples from the environment, including rivers, Antarctic snow, and biogas plants, have been collected to identify various types of MPs such as polypropylene (PP), polyethylene (PE), polystyrene (PS), and polyethylene terephthalate (PT) (Dumichen et al., 2017; Aves et al., 2022). The generation of MPs is not only limited to their degradative by-products but also emerged from clothing microfibres (Hernandez et al., 2017; Galvao et al., 2020), indoor dust (Zhang et al., 2020), cosmetics (facial scrubs) (Napper et al., 2015), tap water (Tong et al., 2020), and seafood (MPs accumulation) (Leung et al., 2021).

MPs affect the normal functioning of the organisms and may cause several organ-specific toxicities such as neuronal, digestive, reproductive, and developmental toxicity (Yin et al., 2021). Compromised sperm quality in men and infertility problems in women have been reported among plastic industry workers. (Jelnes, 1988; Hougaard et al., 2009). Indeed, micro- and nano-particles of plastics may pose more risk to the reproductive system. Various studies have been conducted on animals in order to understand the effect of MPs on male and female fertility (Hou B. et al., 2021; Haddadi et al., 2022; Wei et al., 2022). Moreover, MPs may also affect the growth of offspring when the mother is exposed for a longer duration, suggesting the detrimental effects of MPs on development and growth (Luo et al., 2019; Wang et al., 2019; Hu et al., 2021). Therefore, further research studies are required to understand the in-depth biological effects of MPs on the reproductive and development process, as they can affect future generations.

## Reproductive toxic effects of MPs exposure

**FIGURE 1:**
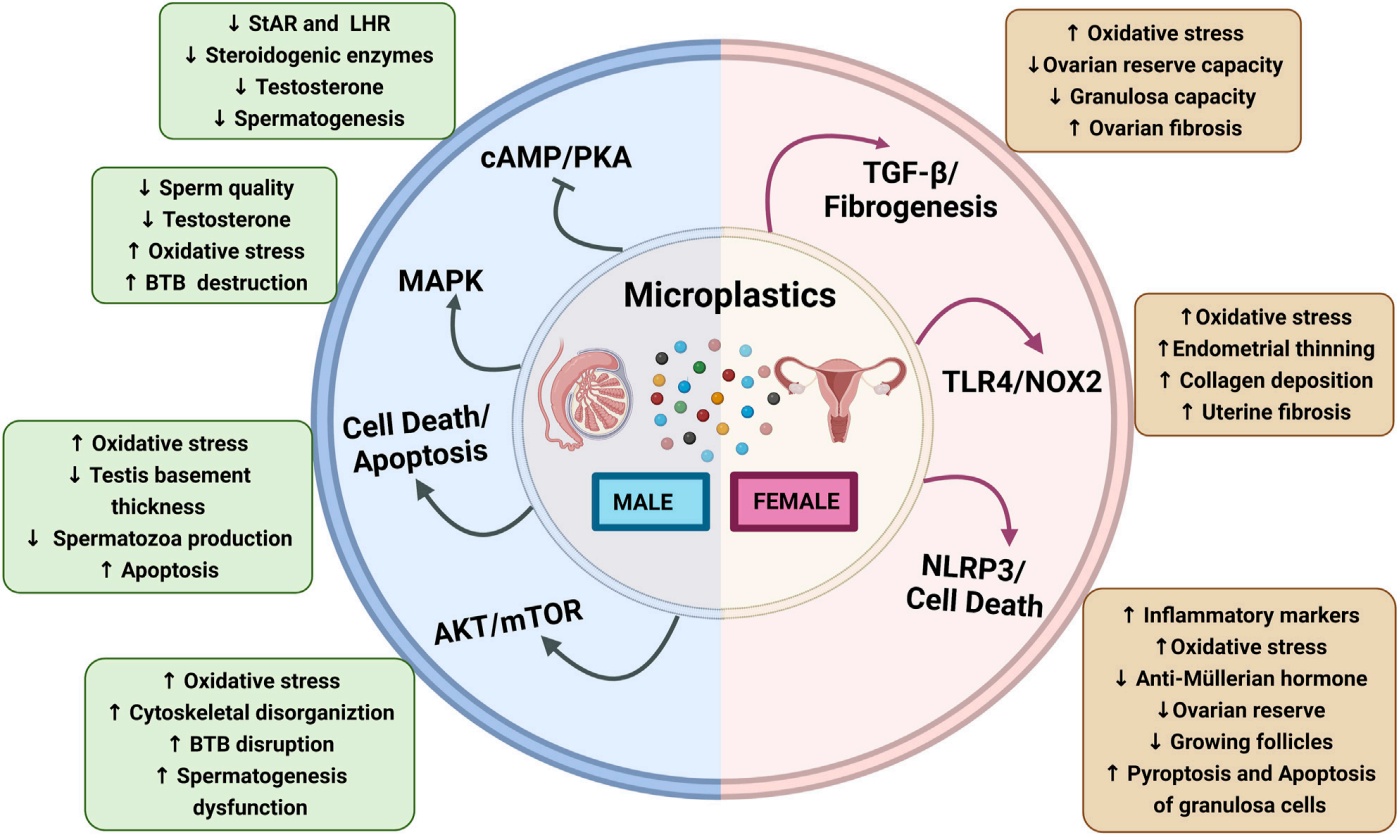
Schematic diagram showing microplastics affecting male and female reproductive functions: Microplastics affecting male reproductive functions *via* the activation of different signaling pathways, Akt/mTOR, apoptosis, MAPK, and inhibition of cAMP/PK3 that results in increased oxidative stress, BTB disruption, and spermatogenesis dysfunction and decrease in steroidogenic enzymes and LHR downregulation. Microplastics affecting the female reproductive functions altered by the activation of NLRP3, TLR4/NOX2, and TGF-β/fibrogenesis that results in increased oxidative stress, endometrial thinning, collagen deposition, inflammatory markers, and pyroptosis and apoptosis of granulosa cells while ovarian reserve, growing follicles, granulosa cells capacity, and anti-müllerian hormone levels are decreased. Abbreviations: cAMP, cyclic adenosine monophosphate; AKT, Ak strain transforming; BTB, blood–testes barrier; LHR, luteinizing hormone receptor; MAPK, mitogen-activated protein kinase; mTOR, mammalian target of rapamycin; NOX-2, NADPH oxidase-2; NLRP3, nod-like receptor family pyrin domain containing 3; PKA, protein kinase A; StAR, steroidogenic acute regulatory protein; TGF-β, transforming growth factor-β; TLR4, toll-like receptor-4.

Reproductive toxicity is defined as exposure to any substance that interferes with the normal functioning of male and female reproductive organs, causing the loss of fertility (United Nations Economic Commission for Europe (UNECE), 2011). Continuous exposure to environmental toxicants and pollutants such as MPs can compromise the fertility of males and females (Wei et al., 2022). It has been reported that MPs induce reproductive toxicity in various organisms, including rodents (An et al., 2021; Hou B. et al., 2021) and aquatic species such as oysters (Sussarellu et al., 2016), cladocerans (Jaikumar et al., 2019), *Caenorhabditis elegans* (Chen et al., 2022), and zebrafish (*Danio rerio*) (Qiang and Cheng, 2021). In this section, we have covered the effect of MPs on structural, functional, and hormonal changes in male and female reproductive organs (Figure 1).

### MPs-induced male reproductive toxicity

MPs-induced reproductive dysfunctions have been observed in the aquatic species, which is attributed to the MPs accumulation in reproductive tissues (Issac and Kandasubramanian, 2021; Qiang and Cheng, 2021; Cormier et al., 2022; Liu Y. et al., 2022). Toxicity evaluation of PE-MPs (<400 µm) in freshwater hydra at concentrations of 0.01, 0.02, 0.04, and 0.08 g/ml for 3–96 h have reported morphological (clubbed and disintegrated tentacles) and reproductive (hydranth numbers) changes; however, these changes are non-lethal (Murphy and Quinn, 2018). Furthermore, Qiang et al. have investigated the testicular toxicity of PS-MPs in zebrafish, and exposure to 100 and 1,000 μg/L of MPs has shown increased testicular apoptosis (Qiang and Cheng, 2021). Moreover, the effect of MPs on testis has not been limited to zebrafish but has also gained attention in other organisms. Earthworms (*Eisenia andrei*) exposed to nano-plastics (>100 nm) for 21 days have been shown to reduce male reproductive functions and deformities in sperm cells, such as reduced mature bundle, damaged plasma membranes, and reduced density of sperm and viability of coelomocytes (Kwak and An, 2021). In addition, chronic exposure to PS-MPs (10 µm) for 28 days exhibits a significant decrease in testosterone, spermatogenic cells, and disrupted blood–testis barrier (BTB) integrity in Balb/c mice (Jin et al., 2021). Some dose-response studies have been performed to evaluate the effects of MPs on the male reproductive organs (Park et al., 2020; Ijaz et al., 2021). PS-MPs have been assessed at the concentration of 2, 20, 200, and 2,000 μg/L in SD rats, and the lowest observed adverse effect level (LOAEL) showed at a dose of 20 μg/L, while the highest concentration (2,000 μg/L) has been reported maximum toxicity (Ijaz et al., 2021). Furthermore, at the highest concentration, PS-MPs have been reported to decrease sperm counts, motility, and viability and also reduce the follicle-stimulating hormone (FSH), luteinizing hormone (LH), and testicular and plasma testosterone levels (Ijaz et al., 2021). MPs (4 and 10 µm) exposure to male Balb/c mice for 28 days have disrupted the BTB and caused testicular inflammation *via* the downregulation of BTB-linked proteins (tight junction protein zonula occludens-1, occludin, basal ectoplasmic specializations (ES) protein, N-cadherin and β-catenin, and gap junction protein CX43) in the testis (Wei et al., 2021).

The steroidogenic enzymes P450scc, P450c17, 3β-HSD, and 17β-HSD are involved in testosterone synthesis in Leydig’s cells (Sun et al., 2019). Jin et al. reported that administration of 100 and 1,000 μg/L of PS-MPs (0.5, 4, and 10 μm) in mice with drinking water for 180 days had shown a significant decrease in steroidogenic enzymes and steroidogenic acute regulatory protein (StAR) levels (Jin et al., 2022). MPs may also cause morphological changes in sperm, such as absent and small head and acrosome loss (Jin et al., 2022). A repeated oral toxicity study for 28 days in SD rats has shown that PP-MPs induce lesions in the testis and epididymis at 25 mg/kg/day (Jin et al., 2022). A recent study has reported that exposure to PS-MPs (1%–10% crushed PS disposable plates for 90 days) showed a remarkable decrease in epididymal sperm count, motility, and serum testosterone level in male albino Wistar rats (Ilechukwu et al., 2022). Based on the above mentioned findings from diverse model systems, it is pertinent that MPs exposure adversely affects the male reproductive system (Figure 1). However, more evidence is required to validate the mechanism of MPs toxicity on male fertility and reproductive health. Hence, it is suggested that future mechanistic investigations are urgently needed to understand the MPs-associated reproductive toxicities.

### MPs-induced female reproductive toxicity

The harmful effects of MPs have not been limited to the male reproductive system (D'Angelo and Meccariello, 2021) but have also been shown to adversely affect the female reproductive system (Wei et al., 2022). Although, as compared to male reproductive studies, a limited number of studies are conducted to evaluate the harmful effects on the female reproductive system. MPs-associated female reproductive toxicity has been studied in several species, including zebrafish (Qiang and Cheng, 2021), oysters (Sussarellu et al., 2016), zooplankton (copepods) (Cole et al., 2015), medaka fishes (Yan et al., 2020; Li et al., 2022), mice (Liu Z. et al., 2022; Wei et al., 2022), and rats (An et al., 2021). Here, we have summarized the available studies on MPs exposure in female reproductive cells/organs.

Copepods, a zooplankton species, were subjected to PS-beads (20 μm, 75 MPs/ml) and cultured algae (250 µgCL^−1^) for 24 h and have shown a significant reduction in ingestion, fecundity, and survival rate, though no change in laying out eggs were observed (Cole et al., 2015). PS-MPs (2 and 6 µm) exposure (0.023 mg/L) to oysters for 2 months has reported a decrease in diameter and number of oocytes (Sussarellu et al., 2016). The combined effect of MPs with heavy metals in medaka fishes (*Oryzias melastigma*) has been shown to perturb similar changes such as irregular oocytes, partly adhesion, and empty follicle in the ovaries of female medaka (Yan et al., 2020). Moreover, PS-MPs (5 µm) exposure (0.1 mg/day) for 24–26 days by oral gavage has perturbs folliculogenesis such as disrupted follicles maturation, differentiation, and increased number of atretic and cyst follicles in Wistar rats (Haddadi et al., 2022). In addition, estrous cycle disruption due to exposure to MPs has also been reported in female rats (Haddadi et al., 2022). PS-MPs (5 µm) led to a significant decrease in the duration of the fourth estrus cycle and reduced the duration of the metestrus phase compared to control rats (Haddadi et al., 2022). Further, the continuous exposure to PS-MPs (30 mg/kg) for 35 days impaired the follicles development, quality, and maturation of oocytes in the ovaries of mice (Liu Z. et al., 2022). Furthermore, the accumulation of MPs (0.5 µm) in granulosa cells of rats has been reported to interfere with the normal functioning, growth, and differentiation of oocytes and lead to female reproductive toxicity *via* apoptosis, pyroptosis, and fibrosis (An et al., 2021; Hou J. et al., 2021). Therefore, extensive exposure to MPs aggravates various toxicities such as decreased diameter and number of oocytes, decreased or empty follicles, inflamed ovaries, and reduced ovarian reserve (Yan et al., 2020; Hou J. et al., 2021; Liu Z. et al., 2022).

Overall, these studies suggest that MPs are associated with negative effects on the female reproductive system (Figure 1).

### MPs-induced developmental toxicity

Developmental toxicity is defined as any reversible or irreversible functional or structural alteration caused by environmental insult, diet, and toxic chemicals or physical factors that affect organisms’ normal growth, differentiation, development, or behaviour (Hougaard, 2021). In this section, we have discussed the development and growth effects such as fetal growth, deformities, and death in offspring whose parents are exposed to MPs for an extended period. A recent study investigated four generations (F0, F1, F2, and F3) of developmental effects in *Daphnia magna* with 21 days of exposure to MPs and reported a significantly reduced population growth rate and reproduction (Martins and Guilhermino, 2018). Furthermore, there is a slow recovery up to F3 generation, which accounts for the developmental toxicity in the *Daphnia magna* population (Martins and Guilhermino, 2018). Moreover, a transgenerational study of PS-MPs in the marine medaka (*Oryzias melastigma*) has reported a delayed incubation time and gonads maturation, hatching rate, and body length of offspring at the dose of 20 and 200 mg/L (Wang et al., 2019). Findings of aquatic studies have also been translated into rodents. The results of 90 days of repeated exposure (0.125, 0.5, 2 mg/day) to PE-MPs (40–48 µm) have shown a significant reduction in the number of live births/dam, sex ratio, and pups’ body weight in the Institute of Cancer Research (ICR) mice (Park et al., 2020). In addition, MPs have disrupted maternal-fetal connection in allogenic pregnant mice, as evident by increased embryo resorption rate and decreased number and diameter of uterine arterioles (Hu et al., 2021). This finding indicates that MPs may pose a threat to fetus development. Several studies have reported the developmental and reproductive toxicities of MPs in different species, summarized in Table 1. However, more studies are required to decipher the mechanisms of MP-associated developmental toxicity and how MPs could cross the placental barrier and impact growth *in utero* and postnatal stages.

**TABLE 1:** Summary of experimental studies showing developmental and harmful effects of MPs in different species and their offspring.

S.N	Type of toxicity	Model systems	MPs types	MPs sizes and concentrations used	Reported effects and inferences	Reference
1	Developmental toxicity (postnatal)	*Daphnia magna*	Pristine polymer microspheres	1–5 μm (0.1 mg/L)	Decreased growth, reproduction, and population growth rate led to the extinction of F1 generation	Martins and Guilhermino, (2018)
					MPs deposition was seen until F3 generation	
2	Developmental toxicity (postnatal and prenatal)	Marine medaka (*Oryzias melastigma*)	PS- MPs	10 µm (20 and 200 mg/L)	Delayed incubation time reduced the heart and hatching rate and length of body of the offspring	Wang et al. (2019)
3	Developmental toxicity (postnatal)	Marine medaka (*Oryzias melastigma*)	PS-MPs (Phenanthrene)	10 μm (2–200 μg/L)	Higher dose deposited on the chorion reduced the growth and hatching rate and delayed hatching time. MPs at low dose do not accumulate phenanthrene	Li et al. (2020)
4	Developmental toxicity (prenatal and postnatal), and reproductive toxicity	Marine medaka (O*ryzias melastigma*)	MPs + Phenanthrene	13 μm (200 μg/L)	Exacerbated bradycardia in embryos, causing transgenerational toxicity from mother to offspring	Li et al. (2022)
5	Developmental toxicity (postnatal)	Zebrafish (*Danio rerio*)	Polyamide (PA) MPs	6.37–8.13 μm 200 mg/L	Reduced hatching rate and inhibited musculoskeletal development in zebrafish larvae	Zou et al. (2020)
					Macrophages induced proinflammation, apoptosis, and multi-xenobiotics resistance	
6	Developmental toxicity (postnatal)	Zebrafish (*Danio rerio*)	Pristine PE-MPs (Medium density)	20–60 μm (6.2, 12.5, 25.0, 50.0 and 100 mg/L)	Harmful effects such as bigger swim bladder, increased yolk sac, and reduced hatching rate of larvae	Malafaia et al. (2020)
					Larvae at concentrations of 50 and 100 mg/L MPs showed more significant external morphological changes and higher teratogenic abnormality rates	
7	Developmental toxicity (prenatal and postnatal)	Zebrafish (*Danio rerio*)	Pristine PS- MPs + Butylated hydroxyanisole (BHA)	65 nm to 20 μm, (2 mg/L) and (BHA, 1 mg/L)	MPs aggravate the accumulation of BHA in zebrafish larvae viz. reduced hatching rates, increased malformation rates, and decreased calcified vertebrae	Zhao et al. (2020)
8	Developmental toxicity (prenatal and postnatal)	Zebrafish (*Danio rerio*)	Pristine PE-MPs and spiked with benzo α pyrene (MP-BaP)	20–27 µm (1% w/w in the fish diet)	MPs and MP-BaP 30 and 90 dpf (day post-fertilization) lead to altered growth parameters such as reduced fecundity, egg morphology, and yolk area	Tarasco et al. (2022)
					Impairment in the development of caudal fins and bone quality	
9	Developmental toxicity (prenatal and postnatal)	Zebrafish (*Danio rerio*)	PS- MPs	10 μm (200 particles/mL)	Larvae development deformities, moderate hatching rate, and altered antioxidant and cellular function	De Marco et al. (2022)
10	Developmental (prenatal) and reproductive toxicity	Prawn	PS-MPs	(2 and 20 mg/L)	The quality of testicular germ cells and sex hormones are altered, causing decreased hatching success and survival of F1 larvae. PS-MPs bioaccumulated in different tissues of larvae and decreased immunity due to paternal exposure	Sun et al. (2022)
11	Developmental and (prenatal and postnatal) reproductive toxicity	Mice	PS nanoplastics	100 nm (0.1, 1 and 10 mg/L)	Prenatal and postnatal PS-NPs exposure declines birth and postnatal body weight in offspring	Huang et al. (2022)
					Transgenerational testicular toxicities in offspring (reduced testis weight and sperm counts)	
12	Developmental toxicity (postnatal)	ICR Mice	PS- MPs	0.5 and 5 µm (100 and 1,000 μg/L)	Risk of metabolic disorders in offspring	Luo et al. (2019)
					Intergenerational effects on the F1 offspring	
13	Developmental (prenatal and postnatal) reproductive toxicity	Male and female ICR mice	PE-MPs	40–48 μm (0.125, 0.5, and 2 mg/mouse)	Reduced number of live births/dam, sex ratio, and body weight of pups	Park et al. (2020)
					Immune disruption in the offspring of PE-treated maternal or paternal mice	
14	Developmental toxicity (prenatal)	C57BL/6-mated Balb/c mice (Allogenic mice)	PS-MPs	10 μm (250 μg/mouse)	Increased resorption rate and reduced number and diameter of uterine arterioles	Hu et al. (2021)
					Immunological barrier homeostasis disruption in the peripheral blood, placenta, and spleen	

## Potential signaling pathways involved in MPs-induced male and female reproductive toxicity

So far, MPs and their effects on reproductive and developmental organs have been reported in different model systems, primarily in laboratory experiments. MPs-induced reproductive toxicity, dysfunctions, and impairments in fertility are associated with many signaling pathways (Figure 1).

### Oxidative stress and MAPK signaling pathway

Oxidative stress is a primary mediator in male and female reproductive dysfunctions (Khan et al., 2011; Agarwal et al., 2014; Ahmad et al., 2017). Excessive reactive oxygen species (ROS) generation creates an imbalance between oxidant and antioxidant status, leading to lipid peroxidation, DNA damage, and protein breakdown (Ahmad et al., 2017; Yu et al., 2018). Several experimental studies have confirmed that microplastics cause ROS production, which increases oxidative stress in gonads (An et al., 2021; Kim et al., 2021; Wei et al., 2021). In addition, mitogen-activated protein kinases (MAPK) pathways are activated through different stimuli, viz. chemical agents and UV-induced damage, cytokines, and oxidative stress (Stramucci et al., 2018). Xie et al. reported that MPs exposure activated MAPK signaling *via* oxidative stress in the mouse testis (Xie et al., 2020). Moreover, PS-MPs exposure for 6 weeks induces ROS generation and increases the phosphorylation of p38 and JNK MAPK in the testis of Balb/c mice (Xie et al., 2020).

Nuclear factor erythroid 2-related factor 2(Nrf2) is a critical transcription factor and acts as an antioxidant which is negatively regulated by Kelch-like ECH-associated protein 1 (Keap-1) (Kovac et al., 2015). Li et al. have shown that PS-MPs increase oxidative stress, activate the p38 MAPK, and deplete the nuclear Nrf2 pathway, which leads to poor quantity and quality of sperms and compromised BTB integrity (Li et al., 2021). The integrity of the BTB junction is regulated *via* N-clathrin during internalization, while selective infiltration is regulated by occludin through tight connections of BTB (Mruk and Cheng, 2010; Lie et al., 2013). Interestingly, PS-MPs have been reported to damage BTB and significantly decrease the expressions of connexin-43, claudin, and N-cadherin in rats (Li et al., 2021). In a nutshell, the MAPK-Nrf2 pathway and oxidative stress-associated mechanism seem to be involved in MPs-induced reproductive dysfunctions (Figure 1). However, more investigations are needed to identify the precise role of this signaling in MP-induced reproductive adverse effects.

### Akt and mTOR signaling pathway

Mammalian target of rapamycin (mTOR) plays a vital role in the cellular processes by providing energy and cytoskeletal structure. mTOR forms two complexes, mTORC1 and mTORC2, and exerts opposite physiological functions by binding with raptor and rictor (Jesus et al., 2017; Wang and Zhang, 2019). Moreover, it plays a crucial role in the maintenance and process of spermatogenesis (Jesus et al., 2017). The ribosomal protein S6 (rpS6), a downstream gene of mTORC1, has been reported to disorganize the F-actin, resulting in leaky BTB *via* the rpS6-Akt-MMP-9 signaling pathway (Mok et al., 2014; Mok et al., 2015). Also, rictor helps to develop F-actin organization and maintains the BTB integrity *via* protein kinase C alpha (PKC-α) and gap junctions (Mok et al., 2013). A recent study has shown that PS-MPs trigger ROS-mediated imbalance of mTORC1 and mTORC2 signaling, which alter the expression of actin-related protein 3 (Arp3) and epidermal growth factor receptor pathway substrate 8 (Eps8) actin-binding proteins, eventually disrupting BTB integrity and spermatogenesis (Wei et al., 2021). The current research is still in the infancy phase and requires more studies to validate the role of Akt and mTOR signaling pathways in MPs-associated reproductive toxicity.

### Inflammasome (NLRP3) and fibrotic signaling pathways

NLRP3, NOD-like receptor protein 3, is a multi-protein that acts as the defense mechanism against microorganisms, endogenous damage, and toxic stimuli but is also involved in male and female infertility (de Rivero Vaccari, 2020; Sano et al., 2022). The activation of NLRP3 triggers an apoptotic and inflammatory response by converting pro-caspase-1 to caspase-1 and pro-interleukin-1 (pro-IL-1β) and pro-interleukin-18 (pro-IL-18) into interleukin-1β (IL-1β) and interleukin-18 (IL-18), respectively (Charan et al., 2022). A recent study has ascertained that PS-MPs trigger the NLRP3/caspase-1 signaling pathway by oxidative stress, leading to decreased ovarian reserve in rats (Hou J. et al., 2021). However, limited findings are reported on NLRP3-mediated biological effects of MPs.

Fibrosis is a process of development of connective tissue as a repairing response to injury and affects organ structure and function, including the reproductive organs (Amargant et al., 2020). A recent study has reported that PS-MPs exposure causes fibrosis in ovaries *via* activation of toll-like receptor-4/NADPH oxidase-2 (TLR4/NOX2) signaling (Wu et al., 2022). This study has also reported an increase in oxidative stress, which consequently leads to activation of NOTCH and transforming growth factor-β (TGF-β)-mediated fibrosis in the endometrial epithelial cells and uterus (Wu et al., 2022). Furthermore, PS-MPs have elevated the expression of Wnt/β-catenin, alpha-smooth muscle actin (α-SMA), TGF-β, and fibronectin in ovarian granulosa cells, thereby leading to ovarian fibrosis (An et al., 2021). Therefore, inflammatory and fibrotic signaling might be involved in the MPs-induced reproductive toxicity, particularly in females (Figure 1).

### Cell death and apoptotic signaling pathways

Apoptosis pathways are the most explored in MPs-induced male and female reproductive toxicity (Qiang and Cheng, 2021; Liu Z. et al., 2022). Qiang et al. have reported that MPs induced the caspase-dependent apoptosis in zebrafish testis, which is mediated *via* the upregulation of several caspases and p53 (Qiang and Cheng, 2021). Furthermore, PS-MP exposure for 35 days leads to perturbed mitochondrial membrane potential and increased inflammatory and apoptotic markers (caspase-3 and Bax) that result in ovarian inflammation and poor quality of oocytes in mice (Liu Z. et al., 2022). Hence, these studies suggest that both (intrinsic and extrinsic) apoptotic pathways are involved in MPs-induced cell death and apoptosis in reproductive organs (Figure 1).

### Steroidogenic and endocrine signaling pathways

Testosterone is crucial in spermatogenesis and is secreted by Leydig cells and regulated by LH signaling (Ramaswamy and Weinbauer, 2014). LH binds to its receptor (LHR) at the Leydig cell membrane, which, in turn, increases cAMP and other downstream pathways such as protein kinase A (PKA), StAR, and steroid synthases (Tremblay, 2015). Of note, Jin et al. have reported that chronic exposure to PS-MPs reduce testosterone, LH, and FSH contents in rat serum and downregulate the expression of StAR *via* inhibiting the AC/cAMP/PKA pathway *in vitro* (Jin et al., 2022). However, the effects of MPs on steroidogenic and endocrine signaling pathways are meager.

## Conclusion

MPs have been shown to accumulate not only in various organs in experimental models but also in human organs such as blood, lymph, placenta, meconium, and lungs (Segovia-Mendoza et al., 2020; Amato-Lourenco et al., 2021; Braun et al., 2021; Cobanoglu et al., 2021; Ragusa et al., 2021; Jenner et al., 2022). However, MPs accumulation is not identified in the reproductive organs of humans. Assessment of MPs-associated adverse effects in humans is challenging. The concentration and size of MPs and the duration of exposure used in experimental models might be quite low and/or high compared to human exposure to MPs. However, marine organisms can be one of the indirect sources of MPs accumulation in humans from seafood and other packaged food materials. Thus, more regulations and awareness are required to curb the generation of MPs in industries and landfill sites.

The toxic effects of MPs are mainly studied in rodents and aquatic experimental models, but the implication of these findings to the human population is still debatable. MPs might trigger their adverse effects *via* oxidative stress, apoptosis, inflammatory and fibrotic response, and altering hormonal balance. Other than these findings, the mechanism(s) of MPs toxicities remain largely unknown. The current understanding of MPs-associated reproductive toxicity is limited and is a nascent area of research, which needs future mechanistically focused investigation to understand the harmful effects of MPs on male and female reproductive organs, including the risk of developmental effects.

## Glossary

AC: Adenylyl cyclase
Akt: Ak strain transforming
Arp3: Actin-related protein 3
αSMA: Alpha-smooth muscle actin
Bax: B-cell lymphoma 2–associated X protein
BTB: Blood–testes barrier
3β-HSD: 3-beta hydroxysteroid dehydrogenase
17β-HSD: 17β-hydroxysteroid dehydrogenases
cAMP: Cyclic adenosine monophosphate
DNA: Deoxyribonucleic acid
FSH: Follicle-stimulating hormone
ICR: Institute of Cancer Research
JNK: c-Jun N-terminal kinase
LH: Luteinizing hormone
LHR: Luteinizing hormone receptor
MPs: Microplastics
p38 MAPK: p38 mitogen-activated protein kinase
MMP 9: Matrix metallopeptidase 9
mTOR: Mammalian target of rapamycin
mTORC1: mTOR complex 1
mTORC2: mTOR complex 2
NOX: 2 NADPH oxidase-2
Nrf2: Nuclear factor erythroid 2-related factor 2
NLRP3: Nod-like receptor family pyrin domain containing 3
P450scc: Cholesterol side-chain cleavage enzyme
PE: Polyethylene
PET: Polyethylene terephthalate
PP: Polypropylene
PS: Polystyrene
PKA: Protein kinase A
ROS: Reactive oxygen species
SD: *Sprague Dawley*
rpS6: Ribosomal protein S6
StAR: Steroidogenic acute regulatory protein
TGF β: Transforming growth factor-β
TLR4: Toll-like receptor 4
UV: Ultraviolet
Wnt: Wingless-related integration site.

## References

[B1] Agarwal A. Virk G. Ong C. Du Plessis S. S. (2014). Effect of oxidative stress on male reproduction. World J. Mens. Health 32 (1), 1–17. 10.5534/wjmh.2014.32.1.1 24872947 PMC4026229

[B2] Ahmad G. Almasry M. Dhillon A. S. Abuayyash M. M. Kothandaraman N. Cakar Z. (2017). Overview and sources of reactive oxygen species (ROS) in the reproductive system, in Oxidative stress in human reproduction (Cham: Springer), 1–16.

[B3] Amargant F. Manuel S. L. Tu Q. Parkes W. S. Rivas F. Zhou L. T. (2020). Ovarian stiffness increases with age in the mammalian ovary and depends on collagen and hyaluronan matrices. Aging Cell 19 (11), e13259. 10.1111/acel.13259 33079460 PMC7681059

[B4] Amato-Lourenco L. F. Carvalho-Oliveira R. Junior G. R. Dos Santos Galvao L. Ando R. A. Mauad T. (2021). Presence of airborne microplastics in human lung tissue. J. Hazard. Mat. 416, 126124. 10.1016/j.jhazmat.2021.126124 34492918

[B5] An R. Wang X. Yang L. Zhang J. Wang N. Xu F. (2021). Polystyrene microplastics cause granulosa cells apoptosis and fibrosis in ovary through oxidative stress in rats. Toxicology 449, 152665. 10.1016/j.tox.2020.152665 33359712

[B6] Aves A. Revell L. Gaw S. Ruffell H. Schuddeboom A. Wotherspoon N. (2022). First evidence of microplastics in Antarctic snow. Cryosphere 16, 2127–2145. 10.5194/tc-16-2127-2022

[B7] Avio C. G. Gorbi S. Regoli F. (2017). Plastics and microplastics in the oceans: from emerging pollutants to emerged threat. Mar. Environ. Res. 128, 2–11. 10.1016/j.marenvres.2016.05.012 27233985

[B8] Braun T. Ehrlich L. Henrich W. Koeppel S. Lomako I. Schwabl P. (2021). Detection of microplastic in human placenta and meconium in a clinical setting. Pharmaceutics 13 (7), 921. 10.3390/pharmaceutics13070921 34206212 PMC8308544

[B9] Charan H. V. Dwivedi D. K. Khan S. Jena G. (2022). Mechanisms of NLRP3 inflammasome-mediated hepatic stellate cell activation: therapeutic potential for liver fibrosis. Genes Dis. 10.1016/j.gendis.2021.12.006 PMC10201559 37223529

[B10] Chen H. Yang Y. Wang C. Hua X. Li H. Xie D. (2022). Reproductive toxicity of UV-photodegraded polystyrene microplastics induced by DNA damage-dependent cell apoptosis in *Caenorhabditis elegans* . Sci. Total Environ. 811, 152350. 10.1016/j.scitotenv.2021.152350 34919931

[B11] Cobanoglu H. Belivermis M. Sikdokur E. Kilic O. Cayir A. (2021). Genotoxic and cytotoxic effects of polyethylene microplastics on human peripheral blood lymphocytes. Chemosphere 272, 129805. 10.1016/j.chemosphere.2021.129805 35534956

[B12] Cole M. Lindeque P. Fileman E. Halsband C. Galloway T. S. (2015). The impact of polystyrene microplastics on feeding, function and fecundity in the marine copepod *Calanus helgolandicus* . Environ. Sci. Technol. 49 (2), 1130–1137. 10.1021/es504525u 25563688

[B13] Cormier B. Cachot J. Blanc M. Cabar M. Clérandeau C. Dubocq F. (2022). Environmental microplastics disrupt swimming activity in acute exposure in *Danio rerio* larvae and reduce growth and reproduction success in chronic exposure in *D. rerio* and Oryzias melastigma. Environ. Pollut. 308, 119721. 10.1016/j.envpol.2022.119721 35809711

[B14] D'Angelo S. Meccariello R. (2021). Microplastics: a threat for male fertility. Int. J. Environ. Res. Public Health 18 (5), 2392. 10.3390/ijerph18052392 33804513 PMC7967748

[B15] De Marco G. Conti G. O. Giannetto A. Cappello T. Galati M. Iaria C. (2022). Embryotoxicity of polystyrene microplastics in zebrafish *Danio rerio* . Environ. Res. 208, 112552. 10.1016/j.envres.2021.112552 34929188

[B16] de Rivero Vaccari J. P. (2020). The inflammasome in reproductive biology: a promising target for novel therapies. Front. Endocrinol. 11, 8. 10.3389/fendo.2020.00008 PMC6997205 32047476

[B17] Dumichen E. Eisentraut P. Bannick C. G. Barthel A. K. Senz R. Braun U. (2017). Fast identification of microplastics in complex environmental samples by a thermal degradation method. Chemosphere 174, 572–584. 10.1016/j.chemosphere.2017.02.010 28193590

[B18] Galvao A. Aleixo M. De Pablo H. Lopes C. Raimundo J. (2020). Microplastics in wastewater: microfiber emissions from common household laundry. Environ. Sci. Pollut. Res. Int. 27 (21), 26643–26649. 10.1007/s11356-020-08765-6 32378098

[B19] Haddadi A. Kessabi K. Boughammoura S. Rhouma M. B. Mlouka R. Banni M. (2022). Exposure to microplastics leads to a defective ovarian function and change in cytoskeleton protein expression in rat. Environ. Sci. Pollut. Res. Int. 29, 34594–34606. 10.1007/s11356-021-18218-3 35040070

[B20] Hernandez E. Nowack B. Mitrano D. M. (2017). Polyester textiles as a source of microplastics from households: a mechanistic study to understand microfiber release during washing. Environ. Sci. Technol. 51 (12), 7036–7046. 10.1021/acs.est.7b01750 28537711

[B21] Hou B. Wang F. Liu T. Wang Z. (2021). Reproductive toxicity of polystyrene microplastics: *In vivo* experimental study on testicular toxicity in mice. J. Hazard. Mat. 405, 124028. 10.1016/j.jhazmat.2020.124028 33087287

[B22] Hou J. Lei Z. Cui L. Hou Y. Yang L. An R. (2021). Polystyrene microplastics lead to pyroptosis and apoptosis of ovarian granulosa cells via NLRP3/Caspase-1 signaling pathway in rats. Ecotoxicol. Environ. Saf. 212, 112012. 10.1016/j.ecoenv.2021.112012 33550074

[B23] Hougaard K. S. Hannerz H. Feveile H. Bonde J. P. (2009). Increased incidence of infertility treatment among women working in the plastics industry. Reprod. Toxicol. 27 (2), 186–189. 10.1016/j.reprotox.2009.01.003 19429396

[B24] Hougaard K. S. (2021). Next generation reproductive and developmental Toxicology: crosstalk into the future. Front. Toxicol. 3, 652571. 10.3389/ftox.2021.652571 35295122 PMC8915852

[B25] Hu J. Qin X. Zhang J. Zhu Y. Zeng W. Lin Y. (2021). Polystyrene microplastics disturb maternal-fetal immune balance and cause reproductive toxicity in pregnant mice. Reprod. Toxicol. 106, 42–50. 10.1016/j.reprotox.2021.10.002 34626775

[B26] Huang T. Zhang W. Lin T. Liu S. Sun Z. Liu F. (2022). Maternal exposure to polystyrene nanoplastics during gestation and lactation induces hepatic and testicular toxicity in male mouse offspring. Food Chem. Toxicol. 160, 112803. 10.1016/j.fct.2021.112803 34990788

[B27] Ijaz M. U. Shahzadi S. Samad A. Ehsan N. Ahmed H. Tahir A. (2021). Dose-dependent effect of polystyrene microplastics on the testicular tissues of the male sprague dawley rats. Dose. Response. 19 (2), 15593258211019882. 10.1177/15593258211019882 34158809 PMC8182192

[B28] Ilechukwu I. Ehigiator B. Ben I. Okonkwo C. Olorunfemi O. Modo E. U. (2022). Chronic toxic effects of polystyrene microplastics on reproductive parameters of male rats. Environ. Anal. Health Toxicol. 37 (2), e2022015–e2022010. 10.5620/eaht.2022015 35878923 PMC9314200

[B29] Issac M. N. Kandasubramanian B. (2021). Effect of microplastics in water and aquatic systems. Environ. Sci. Pollut. Res. Int. 28 (16), 19544–19562. 10.1007/s11356-021-13184-2 33655475 PMC7924819

[B30] Jaikumar G. Brun N. R. Vijver M. G. Bosker T. (2019). Reproductive toxicity of primary and secondary microplastics to three cladocerans during chronic exposure. Environ. Pollut. 249, 638–646. 10.1016/j.envpol.2019.03.085 30933761

[B31] Jelnes J. E. (1988). Semen quality in workers producing reinforced plastic. Reprod. Toxicol. 2 (3-4), 209–212. 10.1016/0890-6238(88)90024-x 2980348

[B32] Jenner L. C. Rotchell J. M. Bennett R. T. Cowen M. Tentzeris V. Sadofsky L. R. (2022). Detection of microplastics in human lung tissue using μFTIR spectroscopy. Sci. Total Environ. 831, 154907. 10.1016/j.scitotenv.2022.154907 35364151

[B33] Jesus T. T. Oliveira P. F. Sousa M. Cheng C. Y. Alves M. G. (2017). Mammalian target of rapamycin (mTOR): a central regulator of male fertility? Crit. Rev. Biochem. Mol. Biol. 52 (3), 235–253. 10.1080/10409238.2017.1279120 28124577 PMC5499698

[B34] Jin H. Ma T. Sha X. Liu Z. Zhou Y. Meng X. (2021). Polystyrene microplastics induced male reproductive toxicity in mice. J. Hazard. Mat. 401, 123430. 10.1016/j.jhazmat.2020.123430 32659591

[B35] Jin H. Yan M. Pan C. Liu Z. Sha X. Jiang C. (2022). Chronic exposure to polystyrene microplastics induced male reproductive toxicity and decreased testosterone levels via the LH-mediated LHR/cAMP/PKA/StAR pathway. Part. Fibre Toxicol. 19 (1), 13–17. 10.1186/s12989-022-00453-2 35177090 PMC8851716

[B36] Khan S. Ahmad T. Parekh C. V. Trivedi P. P. Kushwaha S. Jena G. (2011). Investigation on sodium valproate induced germ cell damage, oxidative stress and genotoxicity in male Swiss mice. Reprod. Toxicol. 32 (4), 385–394. 10.1016/j.reprotox.2011.09.007 22001255

[B37] Kim J. H. Yu Y. B. Choi J. H. (2021). Toxic effects on bioaccumulation, hematological parameters, oxidative stress, immune responses and neurotoxicity in fish exposed to microplastics: A review. J. Hazard. Mat. 413, 125423. 10.1016/j.jhazmat.2021.125423 33930961

[B38] Kovac S. Angelova P. R. Holmstrom K. M. Zhang Y. Dinkova-Kostova A. T. Abramov A. Y. (2015). Nrf2 regulates ROS production by mitochondria and NADPH oxidase. Biochim. Biophys. Acta 1850 (4), 794–801. 10.1016/j.bbagen.2014.11.021 25484314 PMC4471129

[B39] Kwak J. I. An Y. J. (2021). Microplastic digestion generates fragmented nanoplastics in soils and damages earthworm spermatogenesis and coelomocyte viability. J. Hazard. Mat. 402, 124034. 10.1016/j.jhazmat.2020.124034 33254833

[B40] Leung M. M. Ho Y. W. Lee C. H. Wang Y. Hu M. Kwok K. W. H. (2021). Improved Raman spectroscopy-based approach to assess microplastics in seafood. Environ. Pollut. 289, 117648. 10.1016/j.envpol.2021.117648 34332172

[B41] Li S. Wang Q. Yu H. Yang L. Sun Y. Xu N. (2021). Polystyrene microplastics induce blood-testis barrier disruption regulated by the MAPK-Nrf2 signaling pathway in rats. Environ. Sci. Pollut. Res. Int. 28 (35), 47921–47931. 10.1007/s11356-021-13911-9 33895957

[B42] Li Y. Wang J. Yang G. Lu L. Zheng Y. Zhang Q. (2020). Low level of polystyrene microplastics decreases early developmental toxicity of phenanthrene on marine medaka (Oryzias melastigma). J. Hazard. Mat. 385, 121586. 10.1016/j.jhazmat.2019.121586 31759759

[B43] Li Y. Yang G. Wang J. Lu L. Li X. Zheng Y. (2022). Microplastics increase the accumulation of phenanthrene in the ovaries of marine medaka (Oryzias melastigma) and its transgenerational toxicity. J. Hazard. Mat. 424, 127754. 10.1016/j.jhazmat.2021.127754 34838364

[B44] Lie P. P. Cheng C. Y. Mruk D. D. (2013). Signalling pathways regulating the blood-testis barrier. Int. J. Biochem. Cell Biol. 45 (3), 621–625. 10.1016/j.biocel.2012.12.009 23262290 PMC3632505

[B45] Liu Y. Zhang J. Zhao H. Cai J. Sultan Y. Fang H. (2022). Effects of polyvinyl chloride microplastics on reproduction, oxidative stress and reproduction and detoxification-related genes in Daphnia magna. Comp. Biochem. Physiol. C. Toxicol. Pharmacol. 254, 109269. 10.1016/j.cbpc.2022.109269 35026397

[B46] Liu Z. Zhuan Q. Zhang L. Meng L. Fu X. Hou Y. (2022). Polystyrene microplastics induced female reproductive toxicity in mice. J. Hazard. Mat. 424, 127629. 10.1016/j.jhazmat.2021.127629 34740508

[B47] Luo T. Zhang Y. Wang C. Y. Wang X. Y. Zhou J. J. Shen M. L. (2019). Maternal exposure to different sizes of polystyrene microplastics during gestation causes metabolic disorders in their offspring. Environ. Pollut. 255, 113122. 10.1016/j.envpol.2019.113122 31520900

[B48] Malafaia G. de Souza A. M. Pereira A. C. Goncalves S. da Costa Araujo A. P. Ribeiro R. X. (2020). Developmental toxicity in zebrafish exposed to polyethylene microplastics under static and semi-static aquatic systems. Sci. Total Environ. 700, 134867. 10.1016/j.scitotenv.2019.134867 31706091

[B49] Martins A. Guilhermino L. (2018). Transgenerational effects and recovery of microplastics exposure in model populations of the freshwater cladoceran Daphnia magna Straus. Sci. Total Environ. 631, 421–428. 10.1016/j.scitotenv.2018.03.054 29529430

[B50] Mok K. W. Chen H. Q. Lee W. M. Cheng C. Y. (2015). rpS6 regulates blood-testis barrier dynamics through arp3-mediated actin microfilament organization in rat sertoli cells. An *in vitro* study. Endocrinology 156 (5), 1900–1913. 10.1210/en.2014-1791 25714812 PMC4398761

[B51] Mok K. W. Mruk D. D. Cheng C. Y. (2014). rpS6 regulates blood-testis barrier dynamics through Akt-mediated effects on MMP-9. J. Cell Sci. 127 (22), 4870–4882. 10.1242/jcs.152231 25217631 PMC4231304

[B52] Mok K. W. Mruk D. D. Lee W. M. Cheng C. Y. (2013). Rictor/mTORC2 regulates blood-testis barrier dynamics via its effects on gap junction communications and actin filament network. FASEB J. 27 (3), 1137–1152. 10.1096/fj.12-212977 23288930 PMC3574279

[B53] Mruk D. D. Cheng C. Y. (2010). Tight junctions in the testis: new perspectives. Philos. Trans. R. Soc. Lond. B Biol. Sci. 365 (1546), 1621–1635. 10.1098/rstb.2010.0010 20403874 PMC2871926

[B54] Murphy F. Quinn B. (2018). The effects of microplastic on freshwater *Hydra attenuata* feeding, morphology & reproduction. Environ. Pollut. 234, 487–494. 10.1016/j.envpol.2017.11.029 29216486

[B55] Napper I. E. Bakir A. Rowland S. J. Thompson R. C. (2015). Characterisation, quantity and sorptive properties of microplastics extracted from cosmetics. Mar. Pollut. Bull. 99 (1-2), 178–185. 10.1016/j.marpolbul.2015.07.029 26234612

[B56] Park E. J. Han J. S. Park E. J. Seong E. Lee G. H. Kim D. W. (2020). Repeated-oral dose toxicity of polyethylene microplastics and the possible implications on reproduction and development of the next generation. Toxicol. Lett. 324, 75–85. 10.1016/j.toxlet.2020.01.008 31954868

[B57] Qiang L. Cheng J. (2021). Exposure to polystyrene microplastics impairs gonads of zebrafish (*Danio rerio*). Chemosphere 263, 128161. 10.1016/j.chemosphere.2020.128161 33297137

[B58] Ragusa A. Svelato A. Santacroce C. Catalano P. Notarstefano V. Carnevali O. (2021). Plasticenta: first evidence of microplastics in human placenta. Environ. Int. 146, 106274. 10.1016/j.envint.2020.106274 33395930

[B59] Ramaswamy S. Weinbauer G. F. (2014). Endocrine control of spermatogenesis: role of FSH and LH/testosterone. Spermatogenesis 4 (2), e996025. 10.1080/21565562.2014.996025 26413400 PMC4581062

[B60] Sano M. Komiyama H. Shinoda R. Ozawa R. Watanabe H. Karasawa T. (2022). NLRP3 inflammasome is involved in testicular inflammation induced by lipopolysaccharide in mice. Am. J. Reprod. Immunol. 87 (4), e13527. 10.1111/aji.13527 35148014

[B61] Segovia-Mendoza M. Nava-Castro K. E. Palacios-Arreola M. I. Garay-Canales C. Morales-Montor J. (2020). How microplastic components influence the immune system and impact on children health: focus on cancer. Birth Defects Res. 112 (17), 1341–1361. 10.1002/bdr2.1779 32767490

[B62] Stramucci L. Pranteda A. Bossi G. (2018). Insights of crosstalk between p53 protein and the MKK3/MKK6/p38 MAPK signaling pathway in cancer. Cancers 10 (5), 131. 10.3390/cancers10050131 PMC5977104 29751559

[B63] Sun J. Wang D. Lin J. Liu Y. Xu L. Lv R. (2019). Icariin protects mouse Leydig cell testosterone synthesis from the adverse effects of di (2-ethylhexyl) phthalate. Toxicol. Appl. Pharmacol. 378, 114612. 10.1016/j.taap.2019.114612 31175881

[B64] Sun S. Jin Y. Luo P. Shi X. (2022). Polystyrene microplastics induced male reproductive toxicity and transgenerational effects in freshwater prawn. Sci. Total Environ. 842, 156820. 10.1016/j.scitotenv.2022.156820 35738382

[B65] Sussarellu R. Suquet M. Thomas Y. Lambert C. Fabioux C. Pernet M. E. J. (2016). Oyster reproduction is affected by exposure to polystyrene microplastics. Proc. Natl. Acad. Sci. U. S. A. 113 (9), 2430–2435. 10.1073/pnas.1519019113 26831072 PMC4780615

[B66] Tarasco M. Gavaia P. J. Bensimon-Brito A. Cordelières F. P. Santos T. Martins G. (2022). Effects of pristine or contaminated polyethylene microplastics on zebrafish development. Chemosphere 303, 135198. 10.1016/j.chemosphere.2022.135198 35660050

[B67] Tong H. Jiang Q. Hu X. Zhong X. (2020). Occurrence and identification of microplastics in tap water from China. Chemosphere 252, 126493. 10.1016/j.chemosphere.2020.126493 32199168

[B68] Tremblay J. J. (2015). Molecular regulation of steroidogenesis in endocrine Leydig cells. Steroids 103, 3–10. 10.1016/j.steroids.2015.08.001 26254606

[B69] United Nations Economic Commission for Europe (UNECE) (2011). “Globally harmonized system of classification and labelling ofChemicals (GHS),” in Part 3. Health and environmental hazards (United Nations, New York/Geneva: Reproductive Toxicity). Chapter 3.7.

[B70] Wang J. Li Y. Lu L. Zheng M. Zhang X. Tian H. (2019). Polystyrene microplastics cause tissue damages, sex-specific reproductive disruption and transgenerational effects in marine medaka (Oryzias melastigma). Environ. Pollut. 254, 113024. 10.1016/j.envpol.2019.113024 31454586

[B71] Wang Y. Zhang H. (2019). Regulation of autophagy by mTOR signaling pathway. Adv. Exp. Med. Biol. 1206, 67–83. 10.1007/978-981-15-0602-4_3 31776980

[B72] Wei Y. Zhou Y. Long C. Wu H. Hong Y. Fu Y. (2021). Polystyrene microplastics disrupt the blood-testis barrier integrity through ROS-Mediated imbalance of mTORC1 and mTORC2. Environ. Pollut. 289, 117904. 10.1016/j.envpol.2021.117904 34371264

[B73] Wei Z. Wang Y. Wang S. Xie J. Han Q. Chen M. (2022). Comparing the effects of polystyrene microplastics exposure on reproduction and fertility in male and female mice. Toxicology 465, 153059. 10.1016/j.tox.2021.153059 34864092

[B74] Wu H. Xu T. Chen T. Liu J. Xu S. (2022). Oxidative stress mediated by the TLR4/NOX2 signalling axis is involved in polystyrene microplastic-induced uterine fibrosis in mice. Sci. Total Environ. 838 (2), 155825. 10.1016/j.scitotenv.2022.155825 35597360

[B75] Xie X. M. Deng T. Duan J. F. Xie J. Yuan J. L. Chen M. Q. (2020). Exposure to polystyrene microplastics causes reproductive toxicity through oxidative stress and activation of the p38 MAPK signaling pathway. Ecotoxicol. Environ. Saf. 190, 110133. 10.1016/j.ecoenv.2019.110133 31896473

[B76] Yan W. Hamid N. Deng S. Jia P. P. Pei D. S. (2020). Individual and combined toxicogenetic effects of microplastics and heavy metals (Cd, Pb, and Zn) perturb gut microbiota homeostasis and gonadal development in marine medaka (Oryzias melastigma). J. Hazard. Mat. 397, 122795. 10.1016/j.jhazmat.2020.122795 32388101

[B77] Yin K. Wang Y. Zhao H. J. Wang D. X. Guo M. H. Mu M. Y. (2021). A comparative review of microplastics and nanoplastics: toxicity hazards on digestive, reproductive and nervous system. Sci. Total Environ. 774, 145758. 10.1016/j.scitotenv.2021.145758

[B78] Yu P. Liu Z. Wu D. Chen M. Lv W. Zhao Y. (2018). Accumulation of polystyrene microplastics in juvenile Eriocheir sinensis and oxidative stress effects in the liver. Aquat. Toxicol. 200, 28–36. 10.1016/j.aquatox.2018.04.015 29709883

[B79] Zhang J. Wang L. Kannan K. (2020). Microplastics in house dust from 12 countries and associated human exposure. Environ. Int. 134, 105314. 10.1016/j.envint.2019.105314 31756678

[B80] Zhao H-J. Xu J-K. Yan Z-H. Ren H-Q. Zhang Y. (2020). Microplastics enhance the developmental toxicity of synthetic phenolic antioxidants by disturbing the thyroid function and metabolism in developing zebrafish. Environ. Int. 140, 105750. 10.1016/j.envint.2020.105750 32361124

[B81] Zou W. Xia M. Jiang K. Cao Z. Zhang X. Hu X. (2020). Photo-oxidative degradation mitigated the developmental toxicity of polyamide microplastics to zebrafish larvae by modulating macrophage-triggered proinflammatory responses and apoptosis. Environ. Sci. Technol. 54 (21), 13888–13898. 10.1021/acs.est.0c05399 33078945

